# Uniportal Robotic-Assisted Tracheal Resection and Reconstruction Under Spontaneous Ventilation

**DOI:** 10.1155/cria/4991280

**Published:** 2025-01-30

**Authors:** Ricardo Eli Guido Guerra, Francina Valezka Bolaños Morales, Oscar Francisco Silva Gómez, Hector Olvera Prado, Sarahí Ibáñez Barzalobre, Mugurel Bosinceanu, Diego Gonzalez-Rivas

**Affiliations:** ^1^Anesthesiology Service, National Institute of Respiratory Diseases Ismael Cosío Villegas, Mexico City, Mexico; ^2^Thoracic Surgery Service, National Institute of Respiratory Diseases Ismael Cosío Villegas, Mexico City, Mexico; ^3^Department of Thoracic Surgery, Memorial Oncological Hospital, Bucharest, Romania; ^4^Department of Thoracic Surgery and Minimally Invasive Thoracic Surgery Unit, Coruña University Hospital, Coruña, Spain; ^5^Department of Thoracic Surgery, Shanghai Pulmonary Hospital, Tongji University School of Medicine, Shanghai, China

**Keywords:** airway management, anesthesia, lidocaine, minimally invasive surgical procedures, robotic surgical procedures, thoracic surgery video-assisted, tracheal neoplasms

## Abstract

**Background:** Tracheal resection and reconstruction for airway tumors are traditionally performed using general anesthesia, tracheal intubation, and thoracotomy. Modern techniques, such as tubeless tracheal surgery and robotic uniportal approaches, offer several advantages including better surgical conditions, reduced postoperative complications, and faster recovery.

**Case Report:** A 42-year-old woman with a tracheal neuroendocrine tumor underwent nonintubated uniportal robotically assisted tracheal resection and reconstruction. Thoracic epidural anesthesia, airway topicalization, and intravenous anesthesia with laryngeal mask airway allowed the procedure to be performed under nonintubated spontaneous ventilation, through a single 3-cm incision. Postoperative recovery was uneventful, with the patient experiencing minimal pain and no nausea or vomiting.

**Conclusions:** Nonintubated uniportal robotically assisted tracheal resection and reconstruction is a feasible, less invasive technique that offers significant benefits in terms of recovery and patient comfort when performed by experienced surgeons.

## 1. Introduction

Tracheal resection and reconstruction as a treatment for resectable airway tumors is commonly performed under general anesthesia, tracheal intubation, and thoracotomy [1]. Many thoracic surgical procedures have evolved toward less invasive techniques. Tubeless tracheal surgery and robotic uniportal approach are modern techniques offering several advantages, such as better surgical conditions, less postoperative nausea, and vomiting, a faster and improved postoperative recovery and outcome, minimizes airway trauma, and less postoperative pain [2, 3].

## 2. Case

**Figure 1:**
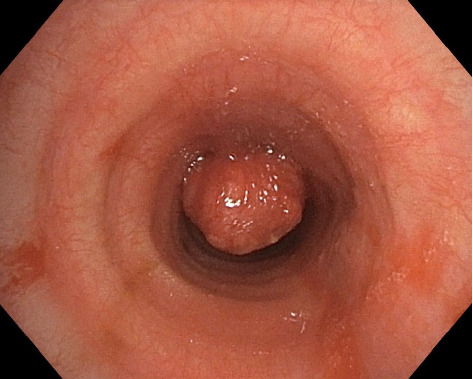
Endotracheal carcinoid tumor at the time of diagnostic approach.

A 42-year-old woman with a tracheal neuroendocrine tumor (Figure 1), macroscopically resected with rigid bronchoscopy, was programmed for a tracheal resection and reconstruction. The patient had no comorbidities and was otherwise healthy.

Upon the patient's arrival in the operating room, standard monitoring procedures were initiated. These included electrocardiogram (ECG), heart rate, pulse oximetry (SpO_2_), end-tidal carbon dioxide (EtCO2), noninvasive blood pressure, and the use of a SEDLine monitor to assess the depth of anesthesia. Prior to the induction of anesthesia, 5 mg of midazolam was administered.

Anesthesia induction was carried out with 40 mg of lidocaine, 150 mcg of fentanyl, 70 mg of propofol, and 30 mg of rocuronium, after which a size 3 iGel laryngeal mask airway was placed. A left radial arterial 20 G catheter was inserted under ultrasound guidance. Bronchoscopy was performed to identify the tracheal lesion, and the airway was topicalized with 2% lidocaine at a dose of 7 mg/kg, targeting the glottis, subglottis, and middle third of the trachea.

The patient was then positioned in the left lateral decubitus position for placement of a thoracic epidural catheter at T6-T7 level. Following a lidocaine/epinephrine test dose of 60 mg/15 mcg, 15 mg of ropivacaine at a 0.75% concentration combined with 50 mcg of fentanyl was administered. Epidural maintenance doses of 15 mg of ropivacaine and 10 mcg of fentanyl were administered hourly. Anesthesia was maintained with continuous infusions of propofol 50–90 mcg/kg/min, dexmedetomidine 0.3–0.5 mcg/kg/hr, and sufentanil 0.001–0.003 mcg/kg/min. A vagus nerve blockade was performed by the surgeon using 4 mL of 0.375% ropivacaine as previously described [4].

The patient's ECG, heart rate, invasive blood pressure, SpO_2_, EtCO_2_, respiratory rate, and Patient State Index were continuously monitored. Neuromuscular blockade was reversed with 3 mg/kg of sugammadex. Spontaneous ventilation was restored, facilitated by pressure support of 0–3 cmH_2_O, and PEEP of 3-4 cmH_2_O, with a fresh gas flow of 2-3 L/min to maintain oxygen saturation above 95%.

**Figure 2:**
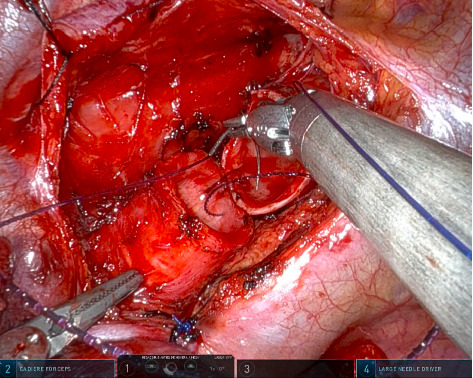
Tubeless field permits excellent visualization during continuous suture of the trachea.

Patient was positioned on the left lateral decubitus position. The da Vinci X system (Intuitive Surgical, Sunnyvale, CA, USA) was then docked to the patient. A single 3 cm skin incision was made at the right 4th intercostal space and thoracic cavity was accessed. The vagus nerve was blocked as described above. Lung collapse was optimal and mediastinal movement minimal. Pleura anterior to trachea was dissected and the vagus nerve was then tacked to the posterior chest wall using prolene sutures. After tracheal dissection, the site of the tumor was localized by using a needle puncture with the help of robotic instruments controlled under bronchoscopic view. The trachea was then transected below and above the tumor and the 7th and 8th tracheal rings were removed. Tracheal reconstruction was performed with barbed suture in a continuous manner (Figure 2). After a negative leak test with saline, pleura was closed, chest drain placed, and closure performed. Total surgical time was 98 min, and tracheal reconstruction time was 19 min.

During tracheal transection, FiO2 was transiently reduced to below 30% with a fresh gas flow of 15–20 L/min during monopolar cutting to prevent ignition. Afterward, FiO_2_ was maintained between 75% and 100% to ensure oxygen saturation above 95% during tracheal reconstruction; no monopolar energy was used during this stage. Endotracheal and cross-field ventilation were prepared as emergency backups in case of hypoxemia, hypercapnia (> 80 mmHg), or pH > 7.15 but were not required. Arterial blood gas analysis at the moment of the reconstruction revealed pH 7.30, pCO_2_ 43 mmHg, pO_2_ 100 mmHg, arterial blood pressure 70–80 mmHg, SpO_2_ 97%–100%, and SEDLine PSI 25–50 during the whole procedure. Emergence was uneventful, LMA was removed, and epidural analgesia was continued along with multimodal analgesia for acute postoperative pain management. The patient reported mild pain and no nausea or vomiting during the postoperative period. The patient was discharged home on the 9th postoperative day. Written informed consent form the patient was obtained for publication.

## 3. Discussion

Tracheal resection and reconstruction is a complex procedure in thoracic surgery. To our knowledge, this is the first case reported in the literature of a nonintubated uniportal robotically assisted thoracic surgery (URATS) tracheal resection and reconstruction. Traditional approaches to intrathoracic tracheal surgery, whether through sternotomy or thoracotomy, are associated with known morbidity. When performed under general anesthesia, the procedure involves incising the trachea, intubating the distal trachea with a sterile endotracheal tube (ETT), and connecting it to a sterile breathing circuit across the surgical field, a method referred to as cross-field ventilation [5]. This technique has inherent challenges, as it requires sequential periods of 2-3 min of apnea to facilitate surgical reconstruction, alongside repeated insertions of the endotracheal tube into the freshly transected trachea. Nonintubated technique offers the advantages of minimizing tracheal manipulation with repeated tube insertion and permits smooth emergence without cough, which is essential to avoid compromising surgical anastomosis [3, 6]. Besides, nonintubated tracheas have been noted to be more flexible, facilitating surgical anastomotic reapproximation [7].

The URATS technique for tracheal reconstruction is a feasible and reliable technique when performed by experienced surgeons. The 3D view and wider range of movements for suturing provided by the robot are advantages of the URATS technique compared with thoracoscopic approaches [2]. The single incision robotic approach which facilitates precision and a minimally invasive approach, in combination with nonintubated tubeless spontaneous ventilation, are two techniques that contribute to an improved recovery profile (one single incision is used instead of 4-5), enhanced surgical conditions and visual field, and reduced surgical times. Over the past 3 decades, there has been a trend toward less invasive methods for tracheal resection and reconstruction, yielding numerous benefits for both patients and surgical teams. However, these techniques are not suitable for all patients and require meticulous patient selection, as significant respiratory or systemic alterations may preclude adequate gas exchange intraoperatively. Factors such as obesity, coagulopathy, ischemia or heart failure, interstitial lung disease, asthma, and COPD are major considerations that may render patients unsuitable for this technique [5, 7]. Currently, no guidelines exist for tracheal resection and reconstruction. It is crucial to identify which patients benefit from tubeless URATS, determine those who should not receive it, establish in which cases it is recommended, and how can it impact the longer term outcomes [3].

## Data Availability

The data used to support the findings of this study are available from the corresponding author upon reasonable request.

## Nomenclature

ETT: Endotracheal tube
COPD: Chronic obstructive pulmonary disease
URATS: Uniportal robotic-assisted thoracic surgery
ECG: Electrocardiogram
EtCO_2_: End expiratory carbon dioxide
LMA: Laryngeal mask airway

## References

[1] Mathisen D. (2018). Distal Tracheal Resection and Reconstruction: State of the Art and Lessons Learned. *Thoracic Surgery Clinics*.

[2] Gonzalez-Rivas D., Bosinceanu M., Manolache V. (2023). Uniportal Fully Robotic-Assisted Sleeve Resections: Surgical Technique and Initial Experience of 30 Cases. *Annals of Cardiothoracic Surgery*.

[3] Grott M., Eichhorn M., Eichhorn F., Schmidt W., Kreuter M., Winter H. (2022). Thoracic Surgery in the Non-Intubated Spontaneously Breathing Patient. *Respiratory Research*.

[4] Hung M. H., Hsu H. H., Chan K. C. (2014). Non-Intubated Thoracoscopic Surgery Using Internal Intercostal Nerve Block, Vagal Block and Targeted Sedation. *European Journal of Cardio-Thoracic Surgery*.

[5] Zhou Y., Liang H., Xu K. (2022). The Strategy of Non-Intubated Spontaneous Ventilation Anesthesia for Upper Tracheal Surgery: A Retrospective Case Series Study. *Translational Lung Cancer Research*.

[6] Jiang L., Liu J., Gonzalez-Rivas D. (2018). Thoracoscopic Surgery for Tracheal and Carinal Resection and Reconstruction Under Spontaneous Ventilation. *The Journal of Thoracic and Cardiovascular Surgery*.

[7] Smeltz A. M., Bhatia M., Arora H., Long J., Kumar P. A. (2020). Anesthesia for Resection and Reconstruction of the Trachea and Carina. *Journal of Cardiothoracic and Vascular Anesthesia*.

